# Supplementary material for: Urine resazurin reduction ratio as a biomarker of urinary tract infection in people with neurogenic bladder: A first in human study
Source: PLoS One. 2026 Feb 11;21(2):e0341599. doi: 10.1371/journal.pone.0341599 (PMC12893601; doi:10.1371/journal.pone.0341599)
Supplement: S1 File — Additional pre-clinical materials discussing the methods used to establish the experimental assay and assess assay linearity and precision. (DOCX) [file pone.0341599.s001.docx]

**Supplemental Materials**

**Supplemental Methods**

**Limit of blank.** The Limit of Blank (LoB) for uRRR was determined using 10 replicates of human filter-sterilized urine on two different devices. Measurements were used to determine the 38.5th ordered value (*n* × 95 / 100 + 0.5; *n* = 40, CLSI EP17-A2) of the ranked result in non-parametric analysis. The results <0 were treated as zero values. The 95th percentile was determined by interpolation of the 38th and 39th ranked values where: 0.5 × 38th + 0.5 × 39th. The final LoB is reported in **Table 1** in the **Supplemental Data**.

**Supplemental Table 1: Analytical Sensitivity of the microfluidic system**

|  |  |  |  |  |
| --- | --- | --- | --- | --- |
| **Strain** | **Parameter** | **Sensor 1 (CFU/mL)** | **Sensor 2 (CFU/mL)** | **Assay performance from 2 devices** |
| *E. coli* ATCC 25922 | Limit of Blank (LoB) | 1.00E+01 | 1.00E+01 | 1.00E+01 |
|  | Limit of Detection (LoD) | 1.00E+02 | 1.00E+02 | 1.00E+02 |
|  | Limit of Quantitation (LoQ) | 1.10E+02 | 1.70E+02 | 1.40E+02 |
| *K. pneumoniae* ATCC 35657 | Limit of Blank (LoB) | 1.00E+01 | 1.00E+01 | 1.00E+01 |
|  | Limit of Detection (LoD) | 1.00E+02 | 1.00E+02 | 1.00E+02 |
|  | Limit of Quantitation (LoQ) | 2.25E+02 | 2.95E+02 | 2.60E+02 |
| *E. faecalis* ATCC 29212 | Limit of Blank (LoB) | 1.00E+01 | 1.00E+01 | 1.00E+01 |
|  | Limit of Detection (LoD) | 4.00E+02 | 6.00E+02 | 5.00E+02 |
|  | Limit of Quantitation (LoQ) | 6.80E+02 | 7.40E+02 | 7.10E+02 |
| *S. saprophyticus* ATCC 15305 | Limit of Blank (LoB) | 1.00E+01 | 1.00E+01 | 1.00E+01 |
|  | Limit of Detection (LoD) | 1.00E+02 | 1.00E+02 | 1.00E+02 |
|  | Limit of Quantitation (LoQ) | 1.30E+02 | 1.10E+02 | 1.20E+02 |

**Limits of detection.** The Limits of Detection (LoD) for the reference strains were estimated using five human sterile-filtered urine samples containing low levels of bacteria using calculations outlined in the precision profile approach in b example of CLSI EP17-A2(32) where:

$$LOD=LOB+c_{p}S.D.$$

$$c_{p}=\frac{1.645}{1-\frac{1}{4(N_{TOT}-K)}}$$

*N*_TOT_ is the total number of measurements (number of measurement results per sample *x* number of samples) and *K* is the number of samples.

Measurements and calculations were performed across two devices, and the precision profile was based on data fitted to a second-order polynomial. The results are reported in **Table 1** in the **Supplemental Data**.

**Limits of quantitation.** The Lower Limits of Quantitation (LLoQ) were determined for the reference strains following Example 1 in Appendix D of CLSI EP17-A2.(32) Five samples with low levels of bacteria following LoD were measured in 16 replicates on two devices. Precision, indicated as % CV, was plotted against mean measured concentration (in CFU/mL) and an intercept at 20% CV of the power function regression line was used to estimate the LLoQ. The LLoQ for all strains was defined as the lowest concentration measurable in the laboratory with 20% CV precision. Final values of LoB, LoD, and LoQ were selected to be the greater of the two values between the two tested instruments and are shown in **Table 1** in the **Supplemental Data**.

**Assay linearity.** Assay linearity for uRRR measurements of the reference strains in the clinically relevant range was demonstrated using six samples containing different CFU/mL of bacteria. Samples were prepared by mixing a high (H) concentration spiked urine sample with a low (L) concentration spiked urine sample, each near the extremes of linear ranges for each species. In the first case, shown in **Fig. 1**, the high sample was human filter-sterilized urine spiked with 10^5^ CFU/mL bacteria, while the low sample contained 10^3^ CFU/mL bacteria. The high and low samples were mixed at varying proportions to assess the proposed linear range. Sample measurements were performed on one device with four replicates per concentration. Linearity was assessed using linear regression.

**Figure 1. Analytical sensitivity of uRRR with uropathogenic bacteria.** (A) Dose-response curves of sterile-filtered urine spiked with *Escherichia coli (red)*, *Klebsiella pneumoniae* (green), *Staphylococcus saprophyticus* (blue), or *Enterococcus faecalis* (magenta)*.* (B) Linearity measurements demonstrating the relationship between signal and bacterial concentration in human sterile-filtered urine samples containing different levels of bacteria. Relative fluorescence units (RFU) measure total resazurin reduction over 15 minutes, normalized by the signal from the background matrix to derive uRRR. Shown are the mean of four measurements with standard deviation (s.d.) error bars, and line of best fit generated using linear regression (lr with 95% confidence interval).

Assay linearity demonstrating bacterial recovery was assessed using seven samples containing different CFU/mL of bacteria in the center of the clinically relevant range (10^3^ - 10^5^ CFU/mL). Samples were prepared by mixing a high (H) concentration spiked urine sample with a low (L) concentration spiked urine sample. In this second case, shown in **Table 2** in **Supplemental Data**, the high sample was human filter-sterilized urine spiked with 7.5 × 10^4^ CFU/mL bacteria, while the low sample contained 1.5 × 10^4^ CFU/mL bacteria. The high and low samples were mixed at varying proportions to assess the proposed linear range. Sample measurements were performed on one sensor device with 4 replicates per level. The measurements and dilution schemes are shown in **Table 2** in **Supplemental Data**.

**Supplemental Table 2: Linearity**

| **Table 2: Linearity** | | | | | | |  |  |
| --- | --- | --- | --- | --- | --- | --- | --- | --- |
|  |  |  |  |  |  |  |  |  |
| **Bacteria in urine measurement and recovery from expected concentrations**  **(*E. coli* ATCC 25922)** | | | | | | |  |  |
| **Level** | **M1 x 1000** | **M2 x 1000** | **M3 x 1000** | **M4 x 1000** | **Mean Conc,x 1000 CFU/mL (%CV)** | **Recovery from expected** |  | **Dilution scheme** |
| 1 (L) | 15.8 | 13 | 16.2 | 16.4 | 15.3 (10.9%) | 100% |  | 1 mL L + 0 mL H |
| 2 | 28.9 | 27 | 28.6 | 28.7 | 28.8 (2.3%) | 104% |  | 0.833 mL L + 0.167 mL H |
| 3 | 44.8 | 42.6 | 42.9 | 43 | 43.6 (2.3%) | 110% |  | 0.75 mL L + 0.25 mL H |
| 4 | 63.3 | 52.9 | 60 | 57.4 | 58.2 (7.5%) | 114% |  | 0.5 mL L + 0.5 mL H |
| 5 | 79.7 | 59.2 | 66.9 | 66.8 | 68.3 (12.8%) | 108% |  | 0.25 mL L + 0.75 mL H |
| 6 | 83.6 | 67.6 | 79.8 | 66.5 | 74.1 (10.3%) | 99% |  | 0.167 mL L + 0.833 mL H |
| 7 (H) | 85.3 | 83.8 | 108.6 | 73 | 87.5 (15.2%) | 100% |  | 0 mL L + 1 mL H |
|  | | | | | | |  |  |
|  |  |  |  |  |  |  |  |  |
| **Bacteria in urine measurement and recovery from expected concentrations**  **(*K. pneumoniae* ATCC 35657)** | | | | | | |  |  |
| **Level** | **M1 x 1000** | **M2 x 1000** | **M3 x 1000** | **M4 x 1000** | **Mean Conc,x 1000 CFU/mL (%CV)** | **Recovery from expected** |  | **Dilution scheme** |
| 1 (L) | 14.4 | 13.3 | 14.6 | 14 | 14.1 (4.1%) | 94% |  | 1 mL L + 0 mL H |
| 2 | 27.8 | 27.9 | 27.9 | 28.8 | 28.1 (1.7%) | 112% |  | 0.833 mL L + 0.167 mL H |
| 3 | 42.1 | 44.7 | 42.9 | 43.8 | 43.4 (2.6%) | 145% |  | 0.75 mL L + 0.25 mL H |
| 4 | 54.2 | 59.5 | 54.8 | 63.3 | 58.0 (7.4%) | 129% |  | 0.5 mL L + 0.5 mL H |
| 5 | 59.8 | 73.1 | 59.7 | 63 | 63.9 (9.9%) | 107% |  | 0.25 mL L + 0.75 mL H |
| 6 | 76.3 | 72.6 | 73.4 | 75.3 | 74.4 (2.3%) | 114% |  | 0.167 mL L + 0.833 mL H |
| 7 (H) | 97.8 | 78.8 | 95.6 | 89.4 | 90.4 (9.4%) | 121% |  | 0 mL L + 1 mL H |
|  | | | | | | |  |  |
|  |  |  |  |  |  |  |  |  |
| **Bacteria in urine measurement and recovery from expected concentrations**  **(*E. faecalis* ATCC 29212)** | | | | | | |  |  |
| **Level** | **M1 x 1000** | **M2 x 1000** | **M3 x 1000** | **M4 x 1000** | **Mean Conc,x 1000 CFU/mL (%CV)** | **Recovery from expected** |  | **Dilution scheme** |
| 1 (L) | 14.8 | 16.8 | 14.7 | 14.3 | 15.2 (7.4%) | 101% |  | 1 mL L + 0 mL H |
| 2 | 28.8 | 28.7 | 27.2 | 28.2 | 28.2 (2.6%) | 113% |  | 0.833 mL L + 0.167 mL H |
| 3 | 44.6 | 41.8 | 43.6 | 41.6 | 42.9 (3.4%) | 143% |  | 0.75 mL L + 0.25 mL H |
| 4 | 62.9 | 53.5 | 62.7 | 61.7 | 60.2 (7.5%) | 134% |  | 0.5 mL L + 0.5 mL H |
| 5 | 69.8 | 61 | 64.7 | 60.3 | 64.0 (6.8%) | 107% |  | 0.25 mL L + 0.75 mL H |
| 6 | 76.1 | 77.6 | 76.8 | 78.3 | 77.2 (1.2%) | 119% |  | 0.167 mL L + 0.833 mL H |
| 7 (H) | 85.8 | 89.9 | 110.6 | 89.5 | 94.0 (12.0%) | 125% |  | 0 mL L + 1 mL H |
|  | | | | | | |  |  |
|  |  |  |  |  |  |  |  |  |
| **Bacteria in urine measurement and recovery from expected concentrations**  **(*S. saprophyticus* ATCC 15305)** | | | | | | |  |  |
| **Level** | **M1 x 1000** | **M2 x 1000** | **M3 x 1000** | **M4 x 1000** | **Mean Conc,x 1000 CFU/mL (%CV)** | **Recovery from expected** |  | **Dilution scheme** |
| 1 (L) | 16.1 | 14.9 | 16.6 | 18 | 16.4 (7.8%) | 109% |  | 1 mL L + 0 mL H |
| 2 | 27 | 28.5 | 28.1 | 28.8 | 28.1 (2.8%) | 112% |  | 0.833 mL L + 0.167 mL H |
| 3 | 43.3 | 41.5 | 44.3 | 44.4 | 43.4 (3.1%) | 145% |  | 0.75 mL L + 0.25 mL H |
| 4 | 54.5 | 63.5 | 55 | 60.1 | 58.3 (7.4%) | 130% |  | 0.5 mL L + 0.5 mL H |
| 5 | 75.3 | 68.4 | 64.4 | 67 | 68.8 (6.8%) | 115% |  | 0.25 mL L + 0.75 mL H |
| 6 | 80.4 | 62.4 | 72.9 | 80.1 | 74.0 (11.4%) | 114% |  | 0.167 mL L + 0.833 mL H |
| 7 (H) | 94.2 | 76.3 | 87.4 | 96.7 | 88.7 (10.3%) | 118% |  | 0 mL L + 1 mL H |
|  | | | | | | |  |  |

**Assay precision.** The precision was evaluated considering the time, operator, instrument, and sample type. First, two concentrations of spiked human filter-sterilized urine samples with high and low concentrations of Gram-negative (*E. coli* ATCC 25922) or Gram-positive (*E. faecalis* ATCC 29212) reference strains within the linear portion of both assays were measured in a single site, by two operators measuring on four sensor devices across three days and two replicates each day. This resulted in total of 48 replicate measurements per sample. The coefficient of variation (%CV) was calculated for each sample. The results of the precision analyses are shown in **Table 3** in the **Supplemental Data**.

**Supplemental Table 3: Assay Precision**

|  |  |  |  |  |  |  |  |  |  |
| --- | --- | --- | --- | --- | --- | --- | --- | --- | --- |
|  |  | **Between Day** | | **Between Operator** | | **Between Instrument** | | **Total Precision** | |
| ***E. coli* ATCC 25922, CFU/mL** | | **SD** | **%CV** | **SD** | **%CV** | **SD** | **%CV** | **SD** | **%CV** |
| **High** | 107 | 6.6 | 0.062 | 5.7 | 5.30% | 4.1 | 3.90% | 4.7 | 4.40% |
| **Low** | 21.4 | 1.11 | 0.052 | 1.14 | 5.40% | 1.11 | 5.20% | 1.9 | 9.00% |
|  |  |  |  |  |  |  |  |  |  |
|  |  | **Between Day** | | **Between Operator** | | **Between Instrument** | | **Total Precision** | |
| ***E. faecalis* ATCC 29212, CFU/mL** | | **SD** | **%CV** | **SD** | **%CV** | **SD** | **%CV** | **SD** | **%CV** |
| **High** | 1110 | 29.6 | 2.70% | 25.7 | 2.30% | 17.2 | 1.50% | 26.3 | 2.40% |
| **Low** | 179 | 4.6 | 2.60% | 6.2 | 2.50% | 4.7 | 2.60% | 5.1 | 2.50% |
|  |  |  |  |  |  |  |  |  |  |
|  |  |  |  |  |  |  |  |  |  |
|  | ***E. coli* ATCC 25922** | | ***E. faecalis* ATCC 29212** | |  |  |  |  |  |
| **Sample** | **CFU/mL** | **%CV** | **CFU/mL** | **%CV** | **Average %CV** |  |  |  |  |
| 1 | 111.7 | 10.10% | 469.8 | 11.90% | 11.00% |  |  |  |  |
| 2 | 117 | 11.40% | 464.2 | 8.30% | 9.80% |  |  |  |  |
| 3 | 117.4 | 8.30% | 528.9 | 9.00% | 8.70% |  |  |  |  |
| 4 | 271.2 | 9.90% | 616.3 | 8.60% | 9.30% |  |  |  |  |
| 5 | 124.7 | 12.10% | 566.9 | 14.10% | 13.10% |  |  |  |  |
|  |  |  |  |  |  |  |  |  |  |
|  |  |  |  |  |  |  |  |  |  |
|  | ***E. coli* ATCC 25922** | | ***E. faecalis* ATCC 29212** | |  |  |  |  |  |
| **Sample** | **CFU/L** | **%CV** | **CFU/mL** | **%CV** | **Average %CV** |  |  |  |  |
| 1 | <LoQ | NA | 31.7 | 9.80% | 9.80% |  |  |  |  |
| 2 | <LoQ | NA | 32.4 | 10.00% | 10.00% |  |  |  |  |
| 3 | <LoQ | NA | 29.1 | 12.30% | 12.30% |  |  |  |  |
| 4 | 8.1 | 11.30% | 37.6 | 7.20% | 9.20% |  |  |  |  |
| 5 | <LoQ | NA | 32.9 | 11.70% | 11.70% |  |  |  |  |

**Calibration Curves.** Eight-point calibration curves were generated in human filter-sterilized urine spiked with 0 - 10^6^ CFU/mL of each strain at the noted densities. Experiments were performed in triplicate. The calibration curves were fitted to nonlinear regression curves using a three-parameter least squares fit model. The equation produced R^2^ values of >0.95 for data points obtained within the limits of detection. uRRR fluorescence values corresponding to ≤10^3^ CFU/mL, >10^3^ and <10^5^ CFU/mL, and ≥10^5^ CFU/mL according to the calibration fit were recorded.

**uRRR quantification in participant samples.** A manual buffer exchange apparatus was derived to filter-capture bacteria and resuspend captured bacteria in growth media before conducting the assay. MHB was used to prewash a sterile syringe filter, which captures the bacteria. Bacteria in the urine sample were captured on the syringe filter and washed using fresh MHB to remove excess contaminants. The flow-through from the bacterial capture was used to prepare the patient-specific negative control by pumping fresh MHB through a second sterile filter. The captured bacteria were eluted using fresh MHB, collected, and incubated at 37˚C for 35 minutes. Resazurin was added to the tubes post-incubation, prior to loading the samples into the microfluidic system. Samples are analyzed as described in the methods (**uRRR quantification**).

**Multivariable Comparisons to uRRR results.** The following steps were used to model uRRR results with multivariable data. A Student's *t*-test or Mann-Whitney U-test, depending on normality, was used to compare continuous variables between two groups. To evaluate whether uRRR levels influence outcomes, univariate and multivariate Cox regression were performed. Association between uRRR and uWBC count was assessed by Pearson’s *Χ*^2^test. All *p*-values are two-tailed and differences were considered statistically significant if *P* < 0.05.

**Statistical comparison of uRRR and bacterial growth by SUC (using bacterial counts).** The 3x3 table used to determine uRRR clinical cutoffs was collapsed into a 2x2 table (<10³ versus >10³), to determine sensitivity and specificity. AUC, sensitivity, and specificity values are valid for the evaluation of a marker; however, they do not specify optimal thresholds directly. We used the index of union (IU) (combined area under the ROC curve (AUC), sensitivity, and specificity values) method for threshold discrimination, which defines the optimal threshold as the point minimizing the summation of absolute values of the differences between AUC and sensitivity and AUC and specificity provided that the difference between sensitivity and specificity is minimum.

To find the highest sensitivity and specificity values at the same time, the AUC value was taken as the starting value. In our analysis of RFU values, we estimated an AUC value of 0.8002 (95% CI: 0.6952 to 0.9053). We identified a threshold from the coordinates of ROC whose sensitivity and specificity values were simultaneously close to or equal to 0.8002, which was defined as the optimal threshold. Using this defined threshold, sensitivity, specificity, PPV, and NPV were calculated for uRRR versus SUC detection for clinical samples (*n* = 289). The false negative rate was estimated together with 95% Confidence Intervals (CI).

**Statistical comparison of uRRR and symptom categories.** We model uRRR results versus patient symptoms and symptom categories in SPSS v29.02 by visually examining variable distribution, conducting extensive cleaning, and examining associations using ANOVA for continuous and *Χ*^2^ tests for categorical variables. Additionally, we employed generalized linear, latent and mixed models, and time-to-event models to accommodate and exploit longitudinal, clustered data. Finally, we conducted multivariable modeling to estimate individual contributions of factors to outcomes. To focus on the effect of a variable, X, on outcome Y, summary variables that “roll up” the effect into a single variable were employed.

Propensity score methods, with and without calibration, were used to protect from confounding. Propensity scores were estimated using multivariable logistic regression, in which patient characteristics were the predictors in a model of the odds of being in a UTI risk group. Covariates were selected based on a priori hypothesized associations, rather than using stepwise algorithms or *p*-value cutoffs. The estimated propensity scores ranged from 0 to 1 for each patient in the study population. The propensity scores were each individual’s predicted probability of being in the UTI- group (generated from the logistic regression model). After calculating propensity scores, the various propensity score methods were used to estimate the odds of exhibiting uRRR results associated with higher bacterial density. 1:1 nearest neighbor matching without replacement was performed with a distance of 0.2 standard deviations, where, for each UTI- group patient, 1 comparison individual in one of the other groups was designated as a match. If the closest match had a propensity score greater than 0.2 standard deviations away from the UTI- group patient, the patient was dropped from the analysis. From this, a density plot of propensity scores in the risk groups was constructed as a visual inspection of balance (**Fig. 1** of the **Supplemental Data**). uRRR was then compared in the matched cohort using logistic regression with additional adjustment for baseline variables whose standardized mean difference was greater than 10%, where standardized mean differences of less than 10% between the groups indicate reasonable balance across each variable.

**Supplemental Figure 1:** Distribution of propensity scores for UTI risk groups.

To account for missing data, we used multiple imputation via the multiple imputation by chained equations (MICE) procedure, which can accommodate variables of varying types (continuous or binary) and complexities in thresholding, which exist among the urinalysis, USQNB, and laboratory data used to comprise UTI risk categories. This procedure assumed that the distribution of each variable (uRR test results) with missing data can be modeled on the basis of the other tests plus the diagnostic outcome with linear regression, as it is a continuous variable. By sampling from the posterior predictive distributions of the missing values, a Gibbs sampler converging after five iterations was used to generate multiple imputations. From this distribution, values were sampled and filled in for the missing test result. The imputation procedure was repeated 10 times. Only 34 cases had missing values, thus needing imputation. The summary statistics showing the distributions among the observed and imputed data separately are shown in **Table 4** in the **Supplemental Data**.

**Supplemental Table 4: Data Imputation by Bladder management**

|  |  |  |  |  |  |  |  |  |  |
| --- | --- | --- | --- | --- | --- | --- | --- | --- | --- |
|  | **Observered** | | **Imputed** | | **Combined** | |  |  |  |
| **Bladder Management** | ***n*** | **Percentage** | ***n*** | **Percentage** | ***n*** | **Percentage** |  |  |  |
| V | 108 | 42.35% | 18 | 52.94% | 126 | 43.60% |  |  |  |
| IC | 101 | 39.61% | 7 | 20.59% | 108 | 37.37% |  |  |  |
| IDC | 46 | 18.04% | 9 | 26.47% | 55 | 19.03% |  |  |  |
| Total | 255 | 100.00% | 34 | 100% | 289 | 100% |  |  |  |

After describing distributions, bivariate associations with outcomes were examined. The evaluation of model quality followed criteria outlined by Allison and Centor. Discrimination was examined using ROC analysis. The OC was examined using likelihood ratios, sensitivity, specificity, and positive/negative predictive values, along with their 95% CI.

**Supplemental Results**

**Evaluation of uRRR quantification.** The microfluidic system-based uRRRs were calibrated using the reference strains spiked into human filter-sterilized urine. The uRRR assay demonstrated high sensitivities with Limits of Detection (LoDs) of 10^2^ CFU/mL (*E. coli*, *K. pneumoniae*, and *S. saprophyticus*) and 5 × 10^2^ CFU/mL (*E. faecalis*) (**Table 1, Supplemental Data**). The Limits of Quantitation (LoQ) were calculated according to CLSI EP17-A2 guidelines described in the supplemental methods and were determined to be 1.4 × 10^2^ CFU/mL (*E. coli*), 2.6 × 10^2^ CFU/mL (*K. pneumoniae*), 1.8 × 10^2^ CFU/mL (*S. saprophyticus*), and 7.1 × 10^2^ CFU/mL (*E. faecalis*) when tested in human sterile-filtered urine (**Table 1, Supplemental Data**). To calculate percent recovery, we compared the mean of replicate values at each dilution against the expected value predicted by the dilution scheme (**Table 2, Supplemental Data**). The uRRR assay showed linear results in human filter-sterilized urine, between 1.5 × 10^4^ CFU/mL and 7.5 × 10^4^ CFU/mL for all strains. Lastly, we evaluated assay precision using human filter-sterilized urine containing known spiked concentrations of each strain. Samples were tested across different days, operators, instruments, and cartridge lots and demonstrated within-lab variation below 15% (**Table 3, Supplemental Data**). These results confirm that the assay behaves as designed, is highly reproducible, and precise.
