# Supplementary figures and images for: Urine resazurin reduction ratio as a biomarker of urinary tract infection in people with neurogenic bladder: A first in human study
Source: PLoS One. 2026 Feb 11;21(2):e0341599. doi: 10.1371/journal.pone.0341599 (PMC12893601; doi:10.1371/journal.pone.0341599)

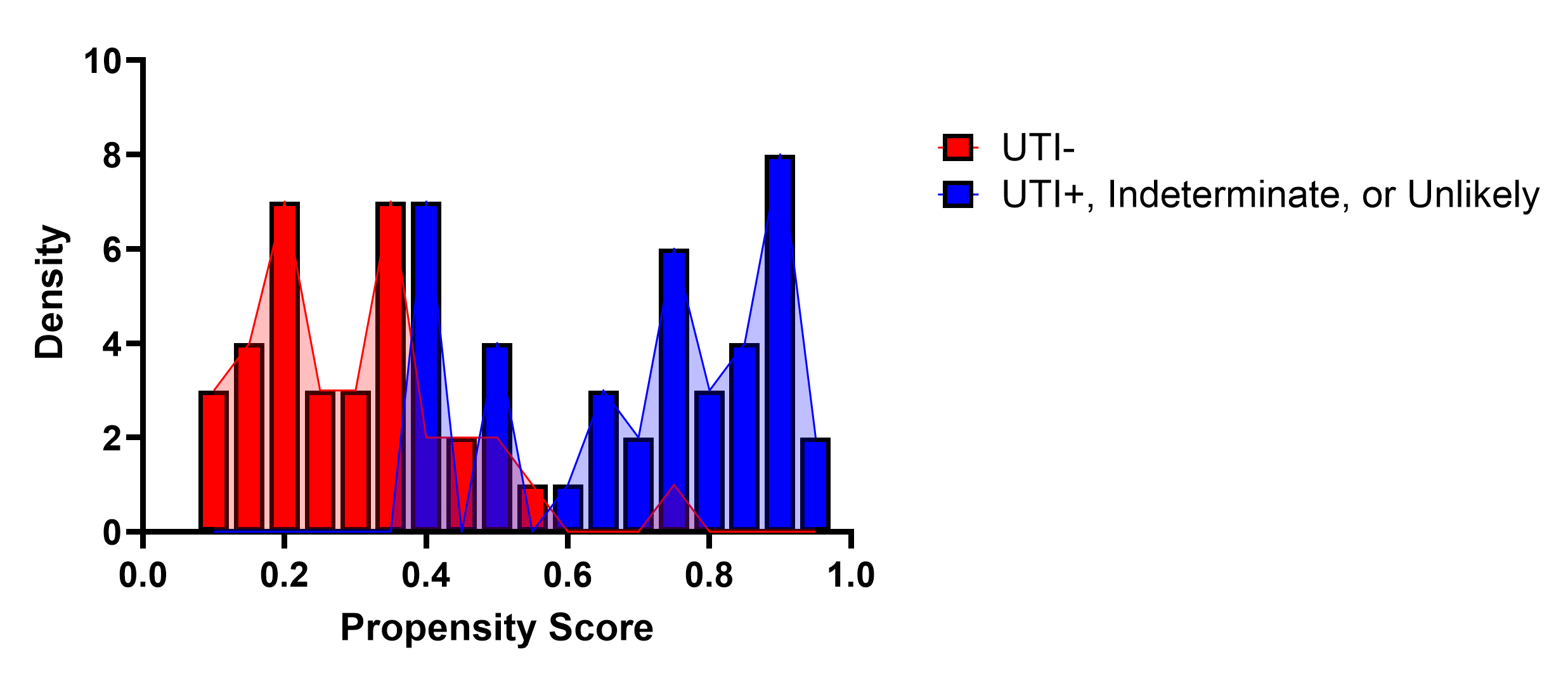

Supplement: S1 Fig — Density plot of propensity scores based on UTI risk groups, constructed as a visual inspection of balance. (TIF) [file pone.0341599.s002.tif]
